# The Impact of Single- and Double-Strand DNA Breaks in Human Spermatozoa on Assisted Reproduction

**DOI:** 10.3390/ijms21113882

**Published:** 2020-05-29

**Authors:** Ashok Agarwal, Cătălina Barbăroșie, Rafael Ambar, Renata Finelli

**Affiliations:** 1 American Center for Reproductive Medicine, Cleveland Clinic, Cleveland, OH 44195, USA; c_barbarosie@hotmail.com (C.B.); rf.ambar@gmail.com (R.A.); finelli.renata@gmail.com (R.F.); 2 Department of Genetics, Faculty of Biology, University of Bucharest, 060101 Bucharest, Romania; 3 Urology Department of Centro Universitario em Saude do ABC/Andrology Group at Ideia Fertil Institute of Human Reproduction, 09015 Santo André, Brazil; 4 Hope Clinic—Human Reproduction, 09060-650 São Paulo, Brazil

**Keywords:** DNA damage, DNA breaks, double-stranded DNA breaks, single-stranded DNA breaks

## Abstract

Several cellular insults can result in sperm DNA fragmentation either on one or both DNA strands. Oxidative damage, premature interruption of the apoptotic process and defects in DNA compaction during spermatogenesis are the main mechanisms that cause DNA breaks in sperm. The two-tailed Comet assay is the only technique that can differentiate single- (SSBs) from double- (DSBs) strand DNA breaks. Increased levels of the phosphorylated isoform of the H2AX histone are directly correlated with DSBs and proposed as a molecular biomarker of DSBs. We have carried out a narrative review on the etiologies associated with SSBs and DSBs in sperm DNA, their association with reproductive outcomes and the mechanisms involved in their repair. Evidence suggests a stronger negative impact of DSBs on reproductive outcomes (fertilization, implantation, miscarriage, pregnancy, and live birth rates) than SSBs, which can be partially overcome by using intracytoplasmic sperm injection (ICSI). In sperm, SSBs are irreversible, whereas DSBs can be repaired by homologous recombination, non-homologous end joining (NHEJ) and alternative NHEJ pathways. Although few studies have been published, further research is warranted to provide a better understanding of the differential effects of sperm SSBs and DSBs on reproductive outcomes as well as the prognostic relevance of DNA breaks discrimination in clinical practice.

## 1. Introduction

**Figure 1:**
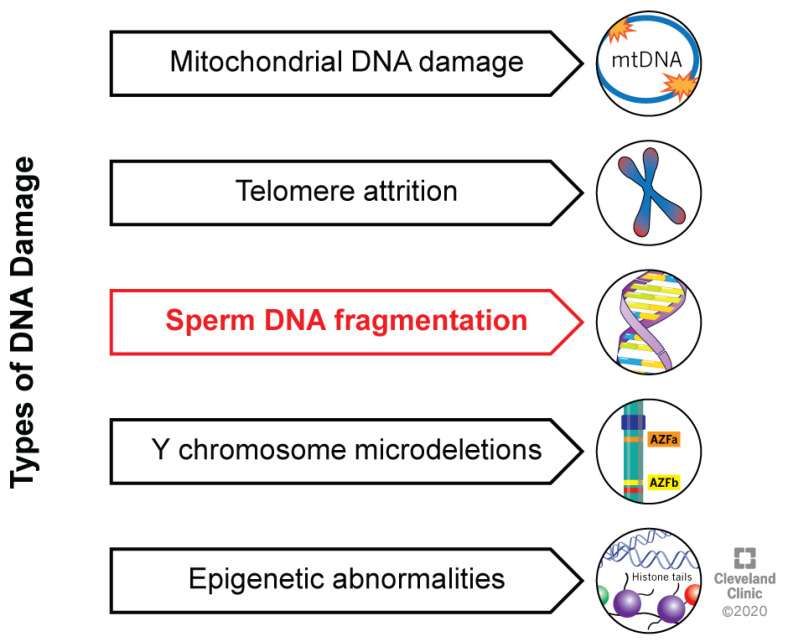
Types of DNA damage. These can include mitochondrial DNA damage, telomere attrition, fragmentation and Y-microdeletions of sperm DNA, and epigenetic abnormalities.

Sperm DNA integrity is crucial for a complete fertilization process, leading to good embryo quality and development, implantation, ongoing pregnancy, and live healthy offspring [1,2,3]. Once fertilization of an oocyte occurs, a male and a female pronucleus appear, containing the genetic material. The fusion between the membranes of pronuclei is followed by DNA replication, driving further zygotic divisions [4]. Zygotic transcription starts at the early stage of development [5], therefore the presence of sperm DNA abnormalities significantly affects embryo development, and the “late paternal effect” due to DNA damage can cause a failure of implantation [4]. Sperm DNA damage is generally categorized as (i) mitochondrial DNA damage, (ii) telomere attrition, (iii) nuclear DNA fragmentation (SDF), (iv) Y-chromosome microdeletions and (v) epigenetic abnormalities (Figure 1) [6].

SDF can affect one or both strands of the DNA helix, resulting in single- (SSBs) or double- (DSBs) strand DNA breaks [7]. In this narrative review, the mechanisms leading to SSBs and DSBs are described, as well as their association with reproductive outcomes and the molecular mechanisms involved in their repair.

## 2. DNA Strand Breaks: Causes and Diagnosis

**Figure 2:**
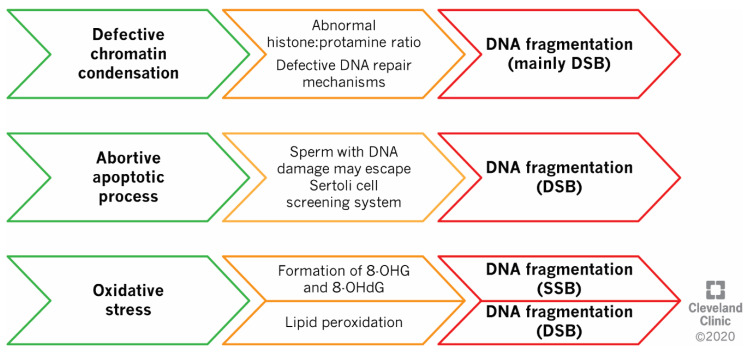
Mechanisms of DNA fragmentation. Defective chromatin condensation and the induction of abortive apoptosis can result in the generation of double-strand DNA breaks (DSBs), while oxidative stress can damage DNA on both single (SSBs) or double-strands (DSBs). Abbreviations: 8-OHG—8-hydroxyguanosine; 8-OHdG—8-hydroxy-2′-deoxyguanosine.

SDF can be induced by three central but interrelated mechanisms, namely defects in sperm compaction, abortive apoptosis, and oxidative stress (Figure 2) [7,8]. 

These mechanisms are strictly interlinked: sperm with defective chromatin compaction are more susceptible to DNA oxidative damage, the latter triggering apoptosis [9,10].

### 2.1. Defects in Sperm DNA Compaction

During spermatogenesis, histones are partially removed and protamines (P1, P2) are assembled in a process named protamination while DNA is tightly compacted side by side in linear arrays [11]. In humans, protamines P1 and P2 are physiologically equally expressed and the compact structure of chromatin is stabilized by the presence of disulfide bonds [12]. Therefore, an altered P1/P2 ratio results in increased susceptibility to DNA damage due to external insults, and consequently, leads to poor reproductive outcomes [13,14]. Moreover, during the histones–protamines replacement, the topoisomerase II works first as an endonuclease to induce relief of the superhelical chromatin structure by promoting transient DSBs [15], and then, it ligates the nicks previously created [16]. If the ATP is not hydrolyzed, topoisomerase II does not dissociate from the DNA and the breaks are not repaired, resulting in permanent damage to sperm DNA [17,18]. 

### 2.2. Abortive Apoptosis

Apoptosis is a crucial process aiming to remove abnormal spermatozoa, in order to maintain an adequate equilibrium between germ cell population and the nursing capacity of Sertoli cells [19]. Sertoli cells screen sperm cells to select those that must undergo apoptosis based on the recognition of the externalized marker phosphatidylserine, and the binding between Fas/FasL molecules expressed on germ and Sertoli cells, respectively [19]. However, the concentration of apoptotic cells can overcome the Sertoli cells’ capability as scavengers, while an unfunctional Fas signaling can allow the apoptotic cells to escape apoptosis, a phenomenon called abortive apoptosis [20,21]. Therefore, the activation of the apoptotic process leads to the generation of DSBs due to the activation of nuclear endonucleases, while the impossibility to complete the process causes the release of damaged sperm in the ejaculate [22,23].

### 2.3. Oxidative Stress

Oxidative stress takes place when the production of reactive oxygen species (ROS) exceeds the antioxidant defenses [24]. Oxidative stress directly damages guanine residues on DNA, resulting in the generation of 8-hydroxy-2′-deoxyguanosine (8-OHdG) and other DNA adducts, such as 1,N6-ethenoadenosine and 1,N6-ethenoguanosine [10,22,25]. Although this process is initially reversible, it can undermine the DNA double-strand architecture and ultimately induce SSBs [22]. Furthermore, an oxidative microenvironment may lead to lipid peroxidation of the sperm membrane, generating radical by-products (i.e., 4-hydroxy-2-nonenal and malondialdehyde) which can activate caspases and endonucleases [9]. Through this mechanism, oxidative stress can indirectly induce apoptosis and cause DSBs [9,22].

### 2.4. Clinical Tests for SDF Assessment

The most common tests for the diagnosis of SDF are the terminal deoxynucleotidyl transferase dUTP nick end labelling (TUNEL), the sperm chromatin structure assay (SCSA), the sperm chromatin dispersion (SCD) test (also known as the Halo test) and the Comet assay (Table 1) [26,27,28].

Although they analyze the same outcome, the results obtained by these different assays are not directly comparable [27]. TUNEL assay analyzes the presence of DNA fragmentation by linking labelled nucleotides at the DNA 3′-OH free-ending [29]. SCSA is based on the metachromatic properties of Acridine Orange (AO) staining [30], where the binding of AO to the native (double-stranded) DNA releases a fluorescence in the green wavelength, while its binding to denatured (single-stranded) DNA is observed in the orange-red spectra. SCD assay is based on the microscopic observation of a “halo” (chromatin dispersion) formed following denaturation [31], whereas a proper halo is not seen when the DNA is damaged. Most of these assays are unable to differentiate between SSBs or DSBs, or the affected DNA region [32]. The two-tailed Comet assay, however, is an exception, as this test can discriminate between SSBs and DSBs by performing the experiment in alkaline denaturing or neutral conditions, respectively [33,34]. In this assay, fragmented DNA molecules are separated electrophoretically and the length of the tail determines the severity of SDF levels. The most recent test introduced for SDF analysis is the immunodetection of γH2AX [28,35]. γH2AX is the phosphorylated form of the histone H2AX and it is found shortly after a DSB takes place, serving as a diagnostic assay only for this type of damage [28].

## 3. Association between DNA SSBs/DSBs and Reproductive Outcomes

Even though the impact of SDF on human reproduction has been widely investigated [36,37], there is scarce evidence on the specific impact of SSBs and DSBs on assisted reproductive technology (ART) outcomes. The association with each outcome is reported in Table 2.

### 3.1. Fertilization and Implantation Rates 

To our knowledge, only four articles have reported the impact of SSBs and DSBs on fertilization and implantation rates [3,38,39,40]. In 2010, Simon et al. used the alkaline Comet assay to analyze SSBs in native semen as well as sperm selected by density gradient centrifugation (DGC) in 360 couples undergoing ART treatment [38]. When IVF was performed (*n* = 230), the authors observed a negative trend of fertilization rate depending on the percentage of sperm with SSBs after DGC separation. Particularly, the fertilization rate significantly decreased when samples with a high percentage of SSBs (SSBs: 61–100%, fertilization rate = 54.4% ± 6.0%) were compared with samples with a low percentage of SSBs (SSBs 0–20%, fertilization rate = 69.9% ± 3.7%; *p* < 0.05) [38]. The negative correlation between fertilization rate and percentage of SSBs was further supported by a later publication when both native semen (*r*^2^: −0.243, *p* = 0.050) and DGC-selected sperm (*r*^2^: −0.276, *p* = 0.025) were used [39]. Conversely, no association was reported for ICSI (*n* = 130) [38]. 

Subsequently, the same group investigated the impact of sperm SSBs on the implantation rate by classifying patients into three categories based on the alkaline Comet assay results: low (0–30%), intermediate (31–70%) and high (71–100%) percentage of sperm with SSBs [3]. A significantly lower implantation rate was reported for both intermediate (55.3%) and high SSB (33.3%) groups in comparison to the low SSB group (65.0%) (*p* < 0.001). However, it is noteworthy that the authors reported the implantation rate for embryos obtained by both IVF and ICSI, without providing differentiated data based on the ART technique used. 

Casanova et al. assessed the impact of SDF on ICSI outcomes by including 196 embryos from 43 infertile couples [40]. They performed two-tailed Comet assay to discriminate between SSBs and DSBs, and subcategorized patients into low or high SSBs and DSBs according to the median value. In agreement with the previous report, fertilization rate was not altered in low and high SSBs groups (fertilization rate = 69% vs. 60%, respectively; *p* = 0.356) and low and high DSBs groups (fertilization rate = 67% vs. 64%, respectively; *p* = 0.701). Furthermore, there was no difference between the low and high SSBs groups regarding the implantation rate (implantation rate = 48% and 24%, respectively; *p* = 0.102) [40]. However, after ICSI, the patient group with a high percentage of sperm DSBs showed a statistically significant delay in embryo development (second polar body extrusion, staged at 4 and 8 cells, morula, and formation of blastocyst; *p* < 0.05) and reduced implantation rate (low DSBs = 52%; high DSBs = 22%; *p* = 0.037) [40].

### 3.2. Miscarriage Rate

Ribas-Maynou et al. analyzed sperm SSBs and DSBs by means of the two-tailed Comet assay in men (*n* = 20) with recurrent pregnancy loss without female factor infertility [41]. These patients showed significantly higher levels of SSBs and DSBs (33.61% ± 15.50% and 84.64% ± 11.28%, respectively) compared with the fertile donor group (*n* = 25) (23.53% ± 10.79% and 44.00% ± 30.18%, respectively) (*p* < 0.01). These data suggest that the presence of sperm DNA DSBs does not impact on the pregnancy rate but increases the risk of a male factor-associated miscarriage. These authors further investigated the power of the alkaline and neutral Comet assay in predicting recurrent miscarriage. They reported a higher specificity (88.0%) and area under the curve (AUC) (0.858) for the neutral Comet assay than the alkaline Comet assay (57.0% and 0.303, respectively), despite a lower sensitivity (neutral Comet = 83.3%; alkaline Comet = 94.4%) [41].

### 3.3. Pregnancy Rate

Simon et al. measured the levels of SSBs with the alkaline Comet assay in native and DGC-selected sperm [38,39,45]. Significantly higher levels of sperm with SSBs were reported in non-pregnant couples who underwent IVF, as compared to pregnant couples, when both native (51.7% ± 23.6% and 39.5% ± 17.9%, *p* = 0.004) and DGC-selected sperm (36.8% ± 21.6% and 26.9% ± 14.6%, *p* = 0.01) were used [38]. Although the percentage of SSBs similarly increased in sperm of non-pregnant couples after ICSI, the results were not significant, suggesting that the ICSI procedure may be able to bypass the presence of DNA damage [38]. These preliminary observations were subsequently supported by the same authors reporting a lower clinical pregnancy rate (44.8%) in couples with high levels of sperm SSBs (SSBs between 71–100%) compared with couples showing lower SSB rate (SSBs between 0–30%, clinical pregnancy rate: 69.7%, *p* = 0.013; SSBs between 31–70%, clinical pregnancy rate: 68.6%, *p* < 0.001) [3]. Pregnancy rate reported after ICSI (60.7%, 82/135 couples) was higher than IVF (52.5%, 42/80 couples), although the difference was not statistically significant [3]. Furthermore, the authors had set clinical sperm SSBs cut-offs for native (56%) and DGC-selected sperm (44%) to predict the clinical pregnancy outcome [38]. They reported a higher sensitivity in IVF and ICSI when DGC-selected sperm (92.3% and 54.6%, respectively) were used in comparison with native semen (82.1% and 47.2%, respectively). However, the specificity of DGC-selected sperm was reportedly lower in IVF and ICSI (34.6% and 63.4%, respectively) than native sperm (49.7% and 68.8%, respectively) [38]. Alkaline Comet was reported to predict clinical pregnancy with relatively moderate sensitivity and specificity (68.75% and 63.46%, respectively) with a cut-off value of 52% [43]. Importantly, the presence of higher SSB rates in native (>52%) and DGC-selected sperm (>46%) was associated with an increased relative risk (RR) of not achieving a clinical pregnancy (RR = 4.75 and 2.16, respectively) [39]. 

Regarding the presence of DSBs, they were determined by flow-cytometric detection of γH2AX in two studies [42,44], where patients were treated by ICSI. Garolla et al. reported a significantly lower percentage of γH2AX-stained sperm in patients who achieved a pregnancy (12.5% ± 8.1%) as compared to those who failed (18.0% ± 12.1%, *p* < 0.05) [42]. These preliminary results were subsequently confirmed in patients undergoing FSH-treatment [44].

When the possibility to achieve a pregnancy was analyzed regarding the presence of sperm with SSBs and DBSs, Ribas-Maynou et al. reported higher values for the alkaline Comet assay (sensitivity: 93.3%; specificity: 90.7%; AUC: 0.965) than neutral Comet assay (sensitivity: 91.1%; specificity: 34.9%; AUC: 0.503) [41]. However, a statistical comparison between the curves was not reported.

### 3.4. Live Birth Rate

The impact of the sperm percentage showing SSBs and DSBs on live birth rate was investigated by Simon et al. and Coban et al., respectively [45,46]. As assessed by the alkaline Comet assay, male partners having <25% sperm with SSBs showed an average live birth rate of 33% following IVF, whereas couples with >50% sperm with SSBs had a much lower live birth rate of 13% (*p* < 0.007) [45]. In contrast, the live birth rate did not differ between ICSI-treated patients with low (<25%, live birth rate = 22%) and high percentage of sperm showing SSBs (>50%, live birth rate = 20.4%) [45]. Recently, the presence of DSBs has been evaluated in IVF patients by detecting γH2AX [46]. The study showed that, in cycles resulting in live birth, sperm levels of γH2AX were significantly lower (17.01% ± 5.68% vs. 23.66% ± 9.68%, *p* = 0.007) than those in sperm from cycles with no birth. Hence, it appears that γH2AX influences the live birth outcome among other studied parameters.

### 3.5. Brief Summary of the above Evidence 

Globally, the current evidence suggests that DSBs have a higher negative impact on reproductive outcomes than SSBs. This may be explained by the separation of paternal and maternal DNA in two different pronuclei during the first stage of embryo development [47], resulting in the absence of a complementary DNA chain to repair the DSBs. Moreover, it has been shown that the cellular cycle can be delayed if a limited percentage of DSBs is present [48]. The influence of sperm DNA damage seems to be reduced when ICSI is employed [38,40,45]. During IVF, the oocyte is incubated with sperm in the same plate, and the fertilization is not facilitated by the embryologist like in ICSI. Therefore, if there is a high rate of DNA damage, fertilization does not occur. On the other hand, in ICSI, the embryologist arbitrarily selects the spermatozoon that appears most suitable based on criteria like motility and morphology. Since a positive correlation between the seminal parameters (motility and normal morphology) and good DNA integrity have been reported in the literature [49,50], it is possible that sperm with low SDF rate are inadvertently selected for ICSI. 

## 4. DNA Repair Mitigation Strategies: Differences between SSBs and DSBs

In male germ cell development and differentiation, DNA damage is repaired until the third week of spermatogenesis [51]. From this stage, sperm DNA begins to be densely compacted and repair mechanisms are downregulated. Therefore, once in the epididymis, acquired sperm DNA damage cannot be repaired [22] and the oocyte is responsible for the maintenance of sperm genomic integrity and stability. However, after zygote formation, the oocyte can effectively repair paternal SSBs and DSBs when the DNA damage in sperm is less than 8% [7,52].

**Figure 3:**
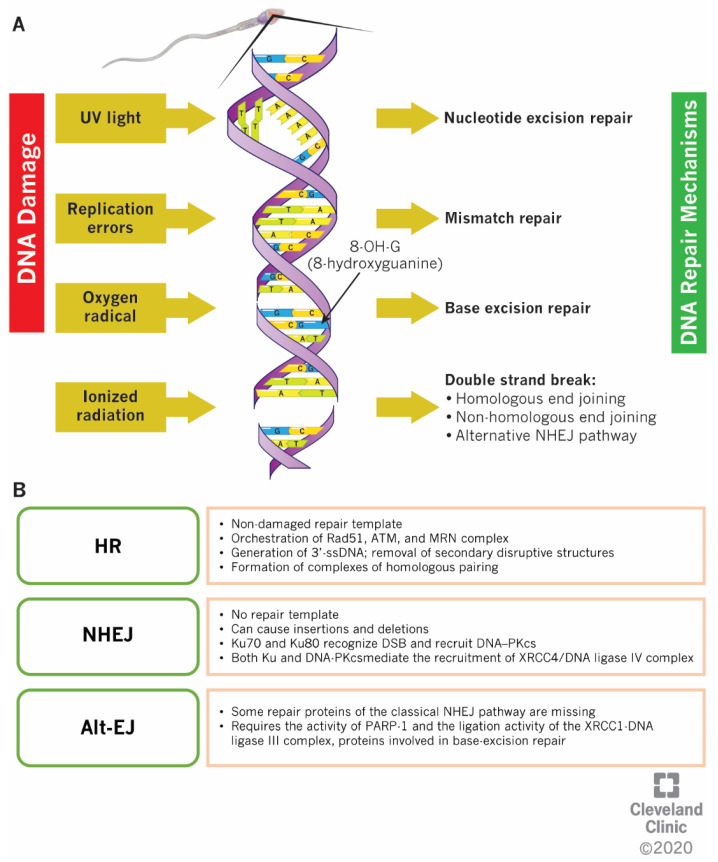
DNA Repair Mechanisms. (**A**) Global overview of the DNA repair mechanisms available in spermatozoa; (**B**) Molecular description of the mechanisms involved in the DNA double-strand breaks repair. Abbreviations: HR homologous recombination; NHEJ – non-homologous end joining; Alt-EJ – alternative NHEJ.

Male germ cells lack molecular SSB repair mechanisms [7]. However, at the stage of spermatids, sperm can rely on the base excision repair (BER) mechanism (Figure 3A) for the removal of the oxidative product 8-OHdG that causes G:C to T:A transversion mutations [53]. 

Identification of altered DNA bases and cleavage of the N-glycosidic bonds are the first steps of the BER pathway [54]. Consequently, an abasic site is generated in the deoxyribose–phosphate complex. This apurinic or apyrimidinic sites are usually cleaved by an endonuclease; however, apyrimidinic endonuclease-1 is not expressed in sperm, thus, the repair does not occur before the S-phase of the first mitotic zygotic division [54].

Cellular mechanisms to repair DSBs include the homologous recombination (HR) and non-homologous end joining (NHEJ) mechanisms (Figure 3B) [55]. DSBs produced during the DNA replication (S-phase of cell cycle) as well as post-replication (G2-phase of cell cycle) are repaired by HR using a non-damaged repair template (the sister chromatid) [56]. During this process, 3′-single-strand DNA ends are generated by removing the secondary disruptive structures, and homologous pairing complexes are formed [57,58,59]. This process is orchestrated upstream by specific molecular components, such as ataxia–telangiectasia-mutated (ATM) and ATM-and Rad3-Related (ATR) kinases. While the ATM is activated specifically by DSBs during the cell cycle, the ATR is involved in the repair of DNA damage arising from the replication process during the S-phase of the cell cycle [60]. Activated ATM phosphorylates downstream effectors, such as BRCA1 and BRCA2. Once BRCA1 is activated, it regulates the activity of the MRE11–RAD50–NBS1 (MRN) molecular complex and indirectly linked with Exonuclease 1, involved in the synthesis of single-strand DNA [61,62]. Both BRCA1 and BRCA2, along with other factors, such as RAD51 and the replication protein A (RPA) on the DNA strands, prevent the DNA degradation by exonucleases [57,63,64]. Contrarily, the NHEJ pathway does not use a template to repair the DSBs and the free DNA junctions are directly linked together after being shortly reduced, therefore, it is characterized by a faster but less accurate DNA repair, where insertions and deletions can occur [55]. There are two types of NHEJ pathways: classical and alternative. In the classical NHEJ pathway, subunits Ku70 and Ku80 form a heterodimer that binds to DSBs and further recruits the DNA-dependent protein kinase catalytic subunit (DNA–PKcs) [65]. DNA–PKcs, in turn, phosphorylates the ARTEMIS nuclease, an enzyme that is involved in the processing of the DNA-free ends [66]. The synergistic action of other enzymes (ligase IV, XRCC4 and Cernunnos-XLF) completes the ligation process [67]. The NHEJ pathway is active along the entire cell cycle [68], but it is particularly relevant in G1-phase, when HR components are absent. In yeast, a competition between both HR and classical NHEJ components has been reported, with MRE11 and CTP1 involved in the removal of Ku proteins from the damaged DNA site and in the dissociation of MRN complex from the DNA free ends, to allow the localization of RPA and to ensure the DNA repair [69,70]. However, in vitro experiments suggest a reduced involvement of Rad51 and other HR-related factors when Ku proteins are not expressed, suggesting a more complex interplay between the pathways [71]. In spermatids, DSBs can be also repaired by the alternative NHEJ pathway (Alt-EJ), which is activated when certain repair proteins of the classical NHEJ pathway are missing, substituted by components of the BER mechanism (i.e., MRN complex, PARP-1 and XRCC1-DNA ligase III) [72,73]. It has been reported that in cells deficient of Ku70, ligase IV, and XRCC4, the Alt-EJ acts as an independent pathway to repair DSBs. The key stages of the Alt-EJ pathway include the recognition of DNA ends, the processing of DSBs, the annealing at microhomologies and polymerase-mediated fill-in, and ligation of DSBs; however, the proteins and mechanisms associated with this pathway are not completely understood [74].

## 5. Conclusions

One or both strands can be damaged in sperm DNA, leading to poor fertility outcomes. Currently, several tests are clinically used to evaluate SDF, however, only the Comet assay can discriminate between SSBs and DSBs. Spermatozoa have the molecular potential to repair DSBs only in the early stages of spermatogenesis, hence, a more significant impact of DSBs on reproductive outcomes has been observed. This can be partially reduced when ICSI is employed. However, further multi-centered clinical studies are necessary to delineate the effect of sperm SSBs and DSBs on reproductive outcomes and the prognostic relevance of the discrimination between these different types of SDF in clinical practice. 

## Figures and Tables

**Table 1:** Techniques that are most frequently used for the assessment of sperm DNA fragmentation.

Test	Principle	Result	Type of Damage Detected
TUNEL	Tagged nucleotides are linked to the DNA break	Sperm with fragmented DNA shows fluorescence	SSBs/DSBs
Comet assay	Fragmented DNA is separated electrophoretically	Shape of comet tail indirectly reflects the severity of DNA damage	Neutral Comet: DSBs Alkaline Comet: mostly SSBs
SCSA	The susceptibility of sperm DNA to denaturation is measured based on the metachromatic properties of acridine orange	Double-stranded DNA fluoresces green, denatured DNA fluoresces orange-red	SSBs/DSBs
SCD test/Halo Test	Chromatin dispersion is microscopically observed after denaturation	DNA integrity results in the generation of a characteristic halo of dispersed DNA loops, while no halo is reported in case of DNA damage	SSBs/DSBs
γH2AX	Antibodies are used to detect γH2AX, a marker of DSB	γH2AX levels correlate with increased levels of DSBs	DSBs

Abbreviations: DSBs—Double-strand breaks; SCD—Sperm Chromatin Dispersion; SCSA—Sperm Chromatin Structure Assay; TUNEL—Terminal deoxynucleotidyl transferase nick end labelling; γH2AX — phospho-histone H2AX; SSBs — Single-strand breaks.

**Table 2:** Summary of studies analyzing the impact of sperm SSBs and DSBs on reproductive outcomes in ART.

Reproductive Outcome	Study Description	Results	Author, Year
Fertilization Rate (FR)	360 patients attending IVF (*n* = 230) and ICSI (*n* = 130); Alkaline Comet assay to evaluate SSBs in the native semen and after DGC	In IVF, FR decreased depending on the percentage of DGC-selected sperm showing SSBs; no difference in ICSI	Simon, 2010 [38]
	75 couples (IVF) and 28 fertile donors; Alkaline Comet assay to assess SSBs in the native semen and after DGC	In IVF, FR was negatively associated to the percentage of sperm with SSBs when both native and DGC sperm were used	Simon, 2011 [39]
	Semen sample used for ICSI was analyzed by two-tailed Comet assay	In ICSI, no difference in FR in case of high percentage of sperm with SSBs and DSBs	Casanovas, 2019 [40]
Implantation Rate (IR)	215 infertile men undergoing IVF/ICSI; samples were classified based on the percentage of SSBs in “low damage”, “intermediate damage” and “high damage”	In the native semen, IR decreased depending on the percentage of sperm with SSBs	Simon, 2014 [3]
	Semen sample used for ICSI was analyzed by two-tailed Comet assay	In ICSI, reduced IR in case of high sperm percentage with DSBs	Casanovas, 2019 [40]
Miscarriage Rate	25 fertile men and 20 patients suffering for recurrent pregnancy loss SDF were analyzed by using two-tailed Comet assay, SCD test and pulsed-field gel electrophoresis (PFGE)	Higher percentage of sperm with SSBs and DSBs is reported in unexplained recurrent pregnancy loss patients than fertile donors	Ribas-Maynou, 2012 [41]
Pregnancy Rate (PR)	360 patients attending IVF (*n* = 230) and ICSI (*n* = 130); Alkaline Comet to evaluate SSBs in the native semen and after DGC	In IVF, non-pregnant couples showed higher percentage of sperm with SSBs than pregnant couples in both native and DGC-selected sperm; cut-offs equal to 56% and 44% for percentage of sperm SSBs in native and DGC-selected semen, respectively, to predict a clinical pregnancy in ART	Simon, 2010 [38]
	75 couples (IVF) and 28 fertile donors; Alkaline Comet assay to assess SSBs in the native semen and after DGC	High percentage of sperm with SSBs (>52% for native semen; >46% for DGC-selected sperm) was associated with decreased pregnancy rate	Simon, 2011 [39]
	25 fertile men and 20 patients suffering for recurrent pregnancy loss DF were analyzed by using two-tailed Comet assay, SCD test and pulsed-field gel electrophoresis (PFGE)	Alkaline Comet assay might better predict pregnancy than neutral Comet assay	Ribas-Maynou, 2012 [41]
	215 infertile men undergoing IVF/ICSI Samples were classified based on the percentage of SSBs in “low damage”, “intermediate damage” and “high damage”	Reduced clinical PR in couples with high percentage of sperm having SSBs	Simon, 2014 [3]
	100 infertile men undergoing ICSI and 61 fertile men DSBs were measured by γH2AX	Reduced percentage of sperm with DSBs in infertile patients who achieved a pregnancy compared to those infertile who failed	Garolla, 2015 [42]
	47 fertile donors and 238 infertile couples; Alkaline Comet assay to evaluate the presence of SSBs	Alkaline Comet predicted clinical pregnancy with moderate sensitivity and specificity at a cut-off value of 52%	Simon, 2017 [43]
	166 infertile male partners of couples undergoing ICSI 84 patients were receiving FSH treatment and 82 refused treatment (controls); DSBs were measured by γH2AX	Infertile patients undergoing FSH-treatment and ICSI showed reduced percentage of sperm with DSBs when the pregnancy was achieved	Garolla, 2017 [44]
Live Birth Rate (LBR)	339 couples attending IVF (*n* = 203) and ICSI (*n* = 136); Alkaline Comet assay to evaluate SSBs in native semen and after DGC	Following IVF, LBR was 33% and 13% in couples with <25% and >50% SSBs, respectively; no difference after ICSI	Simon, 2013 [45]
	60 ART cycles (52 autologous and 8 donors); DSBs assessed by detection of histone γH2AX	In IVF, live birth rate was associated with lower percentage of sperm with DSBs	Coban, 2019 [46]

Abbreviations: ART: assisted reproductive techniques; DGC—density gradient centrifugation; DSBs—double-strand breaks; ICSI—intracytoplasmic sperm injection; IVF—in vitro fertilization; SDF: sperm DNA fragmentation; SSBs—single-strand breaks.

## Abbreviations

ART	Assisted reproductive techniques
DGC	Density gradient centrifugation
DSBs	Double-strand DNA breaks
IVF	In vitro fertilization
SCD	Sperm chromatin dispersion
SCSA	Sperm chromatin structure assay
SSBs	Single-strand DNA breaks
SDF	Sperm DNA fragmentation
TUNEL	Terminal deoxynucleotidyl transferase nick end labelling
